# Body-based therapies and transcranial magnetic stimulation for posttraumatic stress disorder: a proposed integrative approach

**DOI:** 10.3389/fpsyt.2026.1963097

**Published:** 2026-09-17

**Authors:** Lucas Heilbroner, Yousef Abou Areda, Noah S. Philip

**Affiliations:** 1 George Washington University School of Medicine and Health Sciences, Washington, DC, United States; 2 Harvard Medical School, Boston, MA, United States; 3 Center for Neurorestoration and Neurotechnology, Veterans Affairs (VA) Providence Healthcare System and Department of Psychiatry and Human Behavior, Warren Alpert Medical School of Brown University, Providence, RI, United States

**Keywords:** body-based therapy, interoception, neuromodulation, posttraumatic stress disorder, transcranial magnetic stimulation

## Abstract

Posttraumatic stress disorder (PTSD) remains difficult to treat, with 30–50% of patients retaining their diagnosis after first-line trauma-focused psychotherapies. Body-based therapies (BBTs) and transcranial magnetic stimulation (TMS) have each shown preliminary benefit for PTSD but have not yet been studied in combination. To our knowledge, this is the first proposal to integrate these modalities as a combined therapeutic approach. PTSD involves disrupted coordination across multiple levels of neural organization, from cortical regulatory networks to limbic threat circuits and autonomic systems. BBTs are theorized to engage bottom-up processes, including autonomic regulation and interoceptive recalibration, while TMS operates through top-down modulation of prefrontal-limbic circuitry and large-scale network connectivity. This complementarity suggests that a combined approach could engage a broader range of PTSD-related dysfunction than either intervention alone. This article reviews the evidence and proposed mechanisms for each modality, places both within current models of PTSD neurocircuitry, and examines potential pathways of interaction including neuroplasticity priming and autonomic stabilization. Important risks are also addressed, including autonomic destabilization, unknown optimal sequencing, and the open question of whether mechanistically distinct treatments produce additive benefits. The case for combining BBTs and TMS is presented as a hypothesis warranting further investigation.

## 1 Introduction

Posttraumatic stress disorder affects approximately 3.9–8.3% of the population over the course of a lifetime and is associated with profound functional impairment, medical comorbidity, and elevated mortality (1, 2). Although trauma-focused cognitive behavioral therapies, including prolonged exposure and cognitive processing therapy (CPT), remain the first-line treatments, a substantial proportion of patients do not achieve remission. Meta-analyses consistently show that 30–50% of patients retain their PTSD diagnosis after completing first-line treatments (3, 4), and dropout rates from trauma-focused therapies range from 18–36% (5, 6). These limitations have driven the search for novel and adjunctive treatment approaches. Although FDA-approved SSRIs are available for PTSD, pharmacotherapy is outside the scope of this conceptual analysis, which focuses on body-based therapies and neuromodulation.

Two such approaches have emerged from distinct scientific traditions. Body-based therapies (BBTs), rooted in somatic psychology and integrative medicine, emphasize the role of the body in trauma processing and recovery (7, 8). Transcranial magnetic stimulation (TMS), rooted in clinical neuroscience, uses electromagnetic fields to modulate cortical excitability and neural network connectivity (2, 9). To our knowledge, these approaches have not been studied or proposed in combination for PTSD.

PTSD can be understood as involving both top-down difficulties in cognitive and emotional regulation and bottom-up disruptions in bodily arousal, threat detection, and interoceptive processing. BBTs may be especially relevant to the latter, while TMS may be especially relevant to the former. The present article reviews the evidence and proposed mechanisms for BBTs and TMS, situates both approaches within current models of PTSD neurocircuitry, and outlines a framework for future empirical investigation.

## 2 Body-based therapies for posttraumatic stress disorder

### 2.1 Defining modalities

Body-based therapies are a broad group of interventions that use bodily experience as a central part of treatment. They include approaches such as trauma-sensitive yoga, somatic experiencing, sensorimotor psychotherapy, tai chi, and qigong. Although these modalities differ, they share an emphasis on movement, posture, breath, interoception, and physiological regulation (7, 8, 10, 11).

### 2.2 Clinical evidence

Across meta-analytic reviews, body-oriented therapies show small-to-moderate improvements in PTSD symptoms, although findings vary by intervention type, study design, outcome measure, and risk of bias (12). Yoga has the strongest evidence base within this category, including trials of trauma-sensitive yoga in chronic and military sexual trauma-related PTSD.

In a randomized controlled trial (RCT) of 64 women with chronic, treatment-resistant PTSD, van der Kolk et al. (10) found that 52% of participants assigned to trauma-informed yoga no longer met diagnostic criteria for PTSD after 10 weeks, compared with 21% of participants assigned to a health education control condition. The yoga group also demonstrated a large within-group reduction in symptoms (*d* = 1.07). The inclusion of participants who had not responded adequately to previous treatments suggests that body-based approaches may be useful for some individuals whose symptoms persist despite conventional interventions.

More recently, a fully powered RCT comparing Trauma Center Trauma-Sensitive Yoga (TCTSY) to CPT for military sexual trauma-related PTSD found statistically equivalent outcomes between the two treatments, with both producing significant PTSD reductions and similar rates of diagnostic remission, but with higher completion rates in the yoga group, 65.3% vs. 45.8%, *p* = .03 (11). This finding is notable because it positions a body-based therapy as a potential alternative, not merely an adjunct, to a first-line evidence-based psychotherapy.

Beyond yoga, a 2023 RCT demonstrated that brief aerobic exercise immediately following prolonged exposure therapy sessions produced greater reductions in PTSD severity at 6-month follow-up compared to exposure therapy with passive stretching, providing direct evidence that physical movement can augment trauma processing (13).

Despite these promising findings, the evidence base remains limited by modest samples, heterogeneous interventions, varied control conditions, and risk of bias. Some reviews also find stronger effects on self-reported symptoms than clinician-rated outcomes. Overall, BBTs appear clinically useful for some individuals with PTSD, but the evidence does not yet support strong conclusions about comparative efficacy, durability, or specific mechanisms.

### 2.3 Mechanisms of action

BBTs are theorized to operate through several overlapping bottom-up mechanisms, though these mechanisms remain provisional rather than fully established. Broadly, these interventions may help patients shift attention toward bodily experience in a structured and controlled context, potentially changing how internal sensations are noticed, interpreted, and integrated with emotional states.

One proposed mechanism is autonomic regulation. PTSD is often associated with dysregulated arousal, including sympathetic hyperactivation, reduced parasympathetic tone, and states of shutdown or dissociation (14, 15). BBTs may help patients develop greater tolerance of physiological arousal by using movement, breath, posture, and attention to bodily sensation in ways that support regulation rather than avoidance. In this model, body-based practices may help shift patients from chronic defensive states toward states that support greater emotional regulation and social engagement (14–16).

A related mechanism is interoceptive recalibration. Trauma can disrupt interoception, the ability to notice and interpret internal bodily signals, leading either to numbing and disconnection or to heightened somatic vigilance (17, 18). From this perspective, BBTs may function by shifting attention toward internal sensory experience in a controlled context, potentially modifying how bodily signals are interpreted and integrated. Predictive processing models have been used to frame this as a recalibration of interoceptive prediction error signals, although this remains a theoretical extension rather than an established mechanism (19).

Somatic experiencing and related approaches also propose that trauma-related dysregulation reflects incomplete or inhibited defensive responding. In this model, unresolved fight, flight, or freeze responses may contribute to persistent autonomic and motor patterns after trauma. BBTs may address this by using guided attention to movement, posture, kinesthetic experience, and bodily impulses to help patients regain a sense of agency, orientation, and safety (7).

Taken together, these models should be considered complementary but provisional accounts operating at different levels of explanation, rather than a single coherent mechanistic pathway.

## 3 Transcranial magnetic stimulation for PTSD

### 3.1 Modalities and parameters

TMS uses rapidly changing magnetic fields to induce electric currents in targeted cortical regions, modulating neuronal excitability and synaptic plasticity (2). The primary protocols studied in PTSD include high-frequency repetitive TMS, typically 5–20 Hz, which is most commonly applied to the left or right dorsolateral prefrontal cortex (DLPFC); low-frequency (≤1 Hz), which is most commonly applied to the right DLPFC; intermittent theta-burst stimulation (iTBS), a patterned form yielding effects in shorter treatment sessions; and deep TMS, which uses H-coils to stimulate deeper brain structures including the medial prefrontal cortex (9, 20–22).

### 3.2 Clinical evidence

Randomized controlled trials have reported variable outcomes, with some showing meaningful reductions in PTSD symptom severity and others finding modest or nonsignificant differences relative to sham; consequently, meta-analyses have not provided a clear consensus on stimulation target, protocol, and outcome measures (9, 23, 24).

Despite this heterogeneity, several studies have reported encouraging findings. A sham-controlled trial of iTBS in veterans with PTSD found moderate effects that strengthened over time, with a naturalistic follow-up showing that greater accumulated exposure to active stimulation was associated with better outcomes at one year (25, 26). Real-world effectiveness data are also encouraging, including a large multisite cohort in which substantial PTSD symptom reductions were observed across commonly used TMS protocols and were statistically independent of changes in depressive symptoms (21). This independence, together with dose-response findings linking greater active stimulation exposure to superior long-term outcomes and evidence of PTSD-relevant circuit engagement, provides convergent support for a PTSD-specific therapeutic signal that is not readily explained by improvements in depression or by nonspecific treatment effects alone (20, 26, 27).

Combination studies pairing TMS with psychotherapy are especially relevant to the present discussion. One randomized trial found that low-frequency TMS to the right DLPFC delivered before cognitive processing therapy produced greater PTSD symptom reductions than sham stimulation plus therapy, with benefits sustained at follow-up (28). This supports the general possibility that TMS may augment concurrent behavioral treatment. However, another multisite trial found that deep TMS targeting the medial prefrontal cortex combined with brief trauma exposure produced less improvement than sham stimulation with exposure (22). This finding is an important caution: the effects of combining neuromodulation with trauma-related therapeutic work may depend heavily on the type of concurrent therapy or exposure, as other factors such as timing, and perhaps even the patient’s internal state during treatment.

More recent work using personalized functional magnetic resonance imaging (fMRI)-guided targeting of the right DLPFC provided preliminary evidence that TMS can engage threat-related circuitry, including reductions in amygdala threat reactivity (20). Still, this remains an emerging area, and the broader clinical implications are not yet clear.

Overall, the available evidence suggests that TMS reduces PTSD symptoms, but the magnitude and durability of benefit, whether outcomes vary across stimulation parameters, and which patient characteristics might be associated with response remain incompletely defined.

### 3.3 Mechanisms of action

TMS operates primarily through top-down modulation of cortical and network-level systems involved in emotion regulation, threat processing, and learning. Neuroimaging studies have shown that TMS modulates functional connectivity within and between large-scale networks, including the default mode, salience, and central executive networks, with clinical response linked to changes in prefrontal-limbic circuitry, particularly involving the subgenual anterior cingulate cortex (sgACC) and its connections to the DLPFC and amygdala (27, 29, 30). The main neurobiological model of PTSD describes reduced activity in the medial and dorsolateral prefrontal cortex alongside heightened amygdala activity, which is thought to weaken top-down control of fear and amplify threat responses (2, 31, 32).

TMS may exert its effects by shifting connectivity within and between these networks, rather than acting on any single region in isolation. Philip et al. (27) found that clinical improvement after TMS in comorbid PTSD and major depressive disorder (MDD) was associated with changes in sgACC connectivity with the default mode network, DLPFC, insula, and salience network, supporting a network-level model of TMS response rather than a purely focal cortical effect; in another study of iTBS, both between and within-network connectivity was predictive of PTSD symptom reduction (25).

TMS targeting the DLPFC is hypothesized to enhance prefrontal regulation of the amygdala, supporting fear extinction and reducing hyperarousal. The van Rooij et al. study demonstrated that fMRI-guided TMS reduced right amygdala threat reactivity, with clinical symptom differences emerging at follow-up rather than immediately post-treatment, suggesting that mechanistic engagement may precede clinical improvement (20).

TMS may produce effects similar to long-term potentiation or long-term depression, which could in turn support learning-related processes like extinction or the reconsolidation of traumatic memories (33). These plasticity-based effects are especially relevant to PTSD because durable recovery likely requires short-term symptom reduction and changes in how trauma-related memories and threat responses are updated over time.

Overall, TMS may help some patients with PTSD by strengthening prefrontal regulation, altering network connectivity, and supporting plasticity-related learning processes. However, these mechanisms remain incompletely understood, and as indicated by the literature above, it is reasonable to hypothesize that their clinical relevance may depend on TMS variables such as target or protocol, and dose [but see Berlow et al. (21)], as well as on the interaction between stimulation and a patient’s internal state.

## 4 The neurocircuitry of PTSD: a framework for integration

Neuroimaging studies of PTSD have identified alterations across large-scale networks involved in self-processing, threat detection, and cognitive control, including the default mode, salience, and central executive networks. Although findings vary across samples and methods, this literature broadly supports the view that PTSD involves disrupted coordination among regulatory, threat-processing, and self-referential systems (34–37).

Broadly, PTSD is often linked to changes in how brain networks involved in self-related thinking, threat detection, and cognitive control interact. Some studies describe this as greater engagement of the salience network alongside reduced regulatory control from prefrontal parts of the central executive network. Changes in the default mode network have also been reported, especially in relation to autobiographical memory and self-processing, though these findings are less consistent across samples (34–37).

One influential view describes PTSD as a breakdown in coordination across different levels of the brain’s organization, from higher-level cortical control systems to limbic threat circuits and down to brainstem and autonomic regulation. In this account, symptoms do not come from a single faulty region, but from disrupted communication between systems that detect threat, assign meaning to it, and regulate the response (15, 31, 36). Recent causal evidence also supports a circuit-level model of PTSD. Siddiqi et al. (38) identified a lesion-derived PTSD circuit involving the medial prefrontal cortex, amygdala, and anterolateral temporal lobe, and found that reduced connectivity within this circuit after TMS correlated with symptom improvement. This provides convergent support for the idea that PTSD symptoms and treatment response are better understood at the network level than at the level of isolated brain regions.

This framework helps explain the potential complementarity between BBTs and TMS. BBTs may engage bodily, interoceptive, and autonomic processes that are closely tied to bottom-up threat regulation. TMS may more directly engage cortical systems involved in top-down regulation of limbic circuitry. The central question is whether targeting both levels of this system could improve outcomes beyond either approach alone. At present, that remains a theoretical possibility rather than an established clinical claim.

## 5 The case for synergy

### 5.1 Complementary neurobiological targets

The primary rationale for combining BBTs and TMS is that they may engage different aspects of PTSD-related dysregulation. BBTs and TMS may act on different levels of the brain’s organization in PTSD, with BBTs hypothesized to be more closely tied to interoceptive and autonomic processes, and TMS hypothesized to more directly affect cortical control over limbic circuitry (2, 7, 14, 16, 20, 27).

This distinction is clinically useful but conceptual; both approaches likely influence overlapping systems, and additive benefit remains untested. Thus, a combined approach may be best understood as a testable hypothesis before being moved into a therapeutic strategy.

### 5.2 Neuroplasticity priming

One proposed reason for combining BBTs and TMS is that movement-based approaches may influence neuroplasticity-related factors such as brain-derived neurotrophic factor, which could potentially affect how the brain responds to neuromodulation. This idea is indirectly supported by findings from exercise research, although direct evidence specific to BBTs is still limited (13, 39–41).

If movement-based or body-based interventions influence neuroplasticity, they could theoretically alter the brain state in which TMS is delivered. However, this remains speculative. If these priming effects occur, they are likely to be small and dependent on context rather than reliable or uniform. At this point, it is still unclear whether BBTs meaningfully change how the brain responds to subsequent TMS in clinical populations.

### 5.3 Autonomic stabilization as a platform for neuromodulation

Another possible interaction concerns autonomic state during treatment. BBTs may influence physiological arousal and interoceptive awareness (14–17), which could in principle affect how individuals engage with or tolerate subsequent interventions such as TMS. For example, patients who become more able to notice bodily sensations without becoming overwhelmed may be better able to tolerate other therapeutic interventions that activate emotional or threat-related material.

However, this possibility should be framed cautiously. Evidence that autonomic stabilization directly enhances neuromodulation effects is currently limited. Any such relationship is likely indirect and may vary substantially across individuals, particularly given the heterogeneity of autonomic profiles in PTSD. In some patients, body-based work may support regulation; it is easy to imagine that in other patients this approach could lead to increased arousal or even dissociative symptoms if introduced too quickly.

The window of tolerance model offers a useful clinical framework for thinking about how these interventions might interact. Within this model, BBTs may help patients tolerate bodily arousal and internal sensations without becoming overwhelmed, while TMS may support regulatory capacity through cortical-limbic networks (15). However, evidence linking either intervention directly to changes in this framework is limited, so it should be used as a clinical model rather than a demonstrated mechanism of combined treatment.

### 5.4 Precedent from adjacent combination studies

Although no study has directly tested BBTs combined with TMS for PTSD, adjacent literatures provide some support for studying the question. The most directly relevant precedent comes from a randomized clinical trial in which low-frequency TMS to the right DLPFC was delivered immediately before cognitive processing therapy sessions in combat veterans with PTSD (28). Active TMS plus CPT produced greater reductions in PTSD symptom severity compared to sham TMS plus CPT, with benefits sustained at follow-up.

This trial is relevant for the present argument because it demonstrates that TMS can augment a concurrent therapeutic intervention for PTSD when delivered as a preparatory step, the same sequential logic that would underlie a BBT–TMS combination, albeit with a body-based rather than cognitive therapeutic modality. Other combination approaches have also shown promise. Van ‘t Wout-Frank et al. (42) found that tDCS delivered during virtual reality trauma exposure produced greater PTSD symptom reductions than sham stimulation with VR, demonstrating that noninvasive brain stimulation can be paired with a structured experiential intervention to enhance outcomes. This provides further support for the combination of neuromodulatory interventions, however, as reviewed above [e.g., Isserles et al. (22)] the evidence for TMS remains unclear.

This adjacent evidence supports feasibility and plausibility, but it does not establish that BBTs and TMS will be additive or synergistic. The more conservative conclusion is that prior combination studies justify careful empirical testing of BBT–TMS approaches, particularly with attention to sequencing, target selection, patient state, and tolerability.

## 6 Potential risks and counterarguments

### 6.1 Autonomic destabilization

Both BBTs and TMS can influence arousal and physiological state, raising the possibility that individuals may respond differently depending on baseline autonomic regulation and current state during treatment. In some patients, body-based work before or after TMS could support regulation and treatment engagement. In others, it could increase arousal, somatic threat perception, or dissociation, especially if introduced too quickly or without adequate stabilization.

### 6.2 Sequencing and timing

The optimal sequencing of BBTs and TMS is unknown. BBTs may influence autonomic and interoceptive state in ways that affect responsiveness to TMS, and TMS may influence regulatory capacity in ways that affect engagement with BBTs, but these interactions remain speculative. It is not yet clear whether BBTs should precede TMS, follow TMS, occur on separate days, or be delivered concurrently.

Evidence from combined TMS and psychotherapy studies suggests that treatment effects can depend on brain state and context. While data from the study by Kozel et al. (28) indicated that TMS delivered separately from exposure therapy could yield therapeutic effects, the use of script-driven trauma exposure prior to deep TMS produced inferior outcomes compared with sham. This finding is especially important for the present argument because it cautions against assuming that combining neuromodulation with trauma-related activation will automatically improve outcomes.

### 6.3 The question of additivity

It is not yet clear that treatments with different mechanisms produce additive or synergistic benefits. Because BBTs and TMS may affect interacting neural systems, using them together, either at the same time or in sequence, could plausibly lead to added benefit, no difference, or even interference.

Because bottom-up and top-down processes influence each other, changes in one system may also shift how the other responds, and we cannot reliably predict how that will play out. For that reason, the idea that targeting multiple levels of the system will automatically improve outcomes is still an open empirical question rather than something we can assume.

### 6.4 Heterogeneity and methodological challenges

Both BBTs and TMS can vary in how they are delivered, which makes it harder to interpret the existing evidence. Combining them would add another layer of complexity, especially when it comes to choosing appropriate control conditions and separating specific treatment effects from more general, nonspecific ones.

Because of this, properly testing BBT–TMS combinations would likely require well-controlled factorial study designs and adequately powered samples, which may be challenging to achieve in early-stage research. Early studies may therefore need to prioritize feasibility, safety, tolerability, and mechanistic outcomes before moving to larger efficacy trials.

## 7 Future directions

The framework outlined here leads to several testable hypotheses that could be addressed in a staged research program. Early mechanistic studies could examine the immediate neurophysiological effects of combining a single BBT session with TMS, using measures such as cortical excitability, heart rate variability, and neuroimaging markers to determine whether the expected complementary effects occur or whether there is evidence of interference. Heart rate variability may be a particularly informative measure in this context, as heart rate variability features have been shown to predict clinical response to iTBS in PTSD, with higher parasympathetic tone at baseline associated with better outcomes (43). More broadly, the brain-heart axis model describes a pathway connecting frontal and limbic brain regions to the autonomic nervous system and periphery, and proposes that PTSD treatments affecting this circuit, including brain stimulation, may have downstream effects on autonomic regulation and cardiovascular risk (44). Combined BBT–TMS studies could therefore assess whether body-based interventions modify autonomic markers in ways that predict or influence neuromodulation response, providing a testable link between the two treatment modalities.

Early studies should also assess tolerability, dissociation, dropout, and symptom worsening, since these outcomes may be as important as symptom improvement in determining whether the combination is feasible. This is particularly important because both BBTs and TMS may interact with arousal state, trauma cues, and dissociative symptoms in ways that are not yet well understood.

Pilot clinical trials could then use factorial designs to separate the effects of each intervention, with outcomes measured using both clinician-rated and self-report PTSD scales, along with mechanistic endpoints. Choosing specific BBT and TMS protocols for these studies should be grounded in existing evidence. Trauma-sensitive yoga, including Trauma Center Trauma-Sensitive Yoga, which is among the most well-studied yoga-based approaches for PTSD, and low-frequency rTMS targeting the right dorsolateral prefrontal cortex, which has some of the most consistent support among TMS protocols, would be reasonable starting points, with fMRI-guided targeting offering a potential future refinement (9–11, 20).

## 8 Discussion

PTSD can be understood as a disruption in how different levels of the brain coordinate together, from subcortical autonomic and limbic circuits to cortical regulatory networks. Current treatments tend to focus on only part of this system; psychotherapies and TMS are hypothesized to mainly target top-down cortical processes, while BBTs are hypothesized to focus more on bottom-up somatic and autonomic processes. While we recognize this is a very simple heuristic, combining these approaches is therefore an appealing idea and one that has not yet been tested.

The evidence for both BBTs and TMS in PTSD is encouraging but modest, with variability across studies and unclear long-term durability. These limitations may partly reflect the fact that single-modality treatments might not fully engage the broader neural system involved in PTSD. In that sense, addressing only one level of the system may lead to incomplete recovery. A combined approach that targets both levels could, in theory, produce additive effects. At the same time, because bottom-up and top-down systems interact in complex and bidirectional ways, combining interventions could also lead to no added benefit or even interference, which can only be determined empirically.

Given the significant burden of treatment-resistant PTSD, the generally favorable safety profile of both approaches, and the theoretical rationale for integration, this area of research is a promising direction for future study.
